# Targeting microRNAs as key modulators of tumor immune response

**DOI:** 10.1186/s13046-016-0375-2

**Published:** 2016-06-27

**Authors:** Laura Paladini, Linda Fabris, Giulia Bottai, Carlotta Raschioni, George A. Calin, Libero Santarpia

**Affiliations:** Oncology Experimental Therapeutics Unit, IRCCS Humanitas Clinical and Research Institute, Rozzano-Milan, Italy; Department of Experimental Therapeutics, The University of Texas MD Anderson Cancer Center, Houston, TX USA

**Keywords:** MicroRNAs, Cancer, Immune System, Immune-related MicroRNAs, Innate Immunity, Adaptive Immunity, Cancer-Related Immune Response, Anticancer Immunotherapy

## Abstract

The role of immune response is emerging as a key factor in the complex multistep process of cancer. Tumor microenvironment contains different types of immune cells, which contribute to regulate the fine balance between anti and protumor signals. In this context, mechanisms of crosstalk between cancer and immune cells remain to be extensively elucidated. Interestingly, microRNAs (miRNAs) have been demonstrated to function as crucial regulators of immune response in both physiological and pathological conditions. Specifically, different miRNAs have been reported to have a role in controlling the development and the functions of tumor-associated immune cells. This review aims to describe the most important miRNAs acting as critical modulators of immune response in the context of different solid tumors. In particular, we discuss recent studies that have demonstrated the existence of miRNA-mediated mechanisms regulating the recruitment and the activation status of specific tumor-associated immune cells in the tumor microenvironment. Moreover, various miRNAs have been found to target key cancer-related immune pathways, which concur to mediate the secretion of immunosuppressive or immunostimulating factors by cancer or immune cells. Modalities of miRNA exchange and miRNA-based delivery strategies are also discussed. Based on these findings, the modulation of individual or multiple miRNAs has the potential to enhance or inhibit specific immune subpopulations supporting antitumor immune responses, thus contributing to negatively affect tumorigenesis. New miRNA-based strategies can be developed for more effective immunotherapeutic interventions in cancer.

## Background

Local immune response has emerged in the last decade as a key element in the modulation of the multistep process of cancer development [1]. The connection between tumor onset and inflammation has been envisaged after the demonstration that some tumors arise from sites of chronic inflammation. Moreover, not only some tumors are infiltrated by both the innate and adaptive arms of the immune system, but these cells are present even within tumor microenvironment [2]. Immunity has been reported to act both as a pro- or anti-tumorigenic factor depending on the fine-tuned equilibrium between innate and adaptive immune system [3]. In this context, the intercellular communication between cancer and infiltrating immune cells has the main role to modulate this immune response, thus positively influencing tumor development [4]. Among the different molecular players in the field, microRNAs (miRNAs) have been described as small non–coding RNA molecules regulating different physiological and pathological processes, including inflammation and cancer [5–7]. The multifaceted role of miRNAs derives from their mechanism of action, based on post-transcriptional modulation of multiple genes by base-pairing to target messenger RNAs (mRNAs) [8]. Mature miRNAs are 18-24 nucleotides long, and their biogenesis process has been widely studied in the past years. Briefly, primary miRNA (pri-miRNA) transcripts are produced in the nucleus by RNA polymerases II or III and subsequently processed into precursor-miRNAs (pre-miRNAs) by the RNase III domain of endonuclease Drosha, complexed with DCGR8 [8]. Pre-miRNAs are then actively translocated into the cytoplasm by the exportin 5 nuclear protein (EXP5) in complex with Ran-GTP unit. The RNase III endoribonuclease Dicer converts pre-miRNAs into RNA duplexes about 22 nucleotides long, containing one mature miRNA molecule [9]. This strand is finally incorporated into the RNA-induced silencing complex (RISC), which is the functional unit of Argonaute-mediated miRNA/mRNA binding and regulation [10]. Target sequences are usually located in the 3’untranslated region (UTR) of mRNAs and the grade of complementarity determines whether it will be targeted for degradation (total complementarity) or translational repression (partial complementarity) [11].

The growing body of literature demonstrating the importance of miRNAs in tumor onset, progression and response to therapy has defined these molecules as potential cancer biomarkers [12–14]. Different delivery strategies have been developed suggesting novel promising miRNA-based therapeutic approaches in order to overcome limits of current treatment of cancer [15]. Recently, due to the improvements in delivery systems, miRNA-targeting drugs entered into human clinical trials and the first results about the efficacy of this therapy is expected to be presented soon [16, 17]. In the past decades, the importance of miRNAs in the modulation of normal and pathological immune function has been shown in various studies in which deregulation of miRNAs was demonstrated to characterize diseases associated with excessive or uncontrolled inflammation [18]. There is an increasing number of studies directed to investigate miRNA-immunity-cancer connection and all these new data need to be comprehensively understood for novel therapy applications [19, 20]. Specifically, the mechanisms of miRNA exchange between cancer and immune cells have to be further explored and clarified in a tumor context-dependent manner. Exosomes have been demonstrated to represent vehicles of this miRNA transport within tumor microenvironment and recently also in circulation of cancer patients [21]. This review aims to summarize the most important miRNAs as regulators of immune cells (immune-related miRNAs) and to deeply discuss their role in modulating crucial checkpoints of cancer-related immune response in different types of solid tumors. An extensive understanding of effects of these immune-related miRNAs in cancer will very likely allow to identify specific miRNAs as potential targets for cancer immunotherapy.

## The role of immune response in cancer

The human immune system has developed as a complex network of pathways regulating the response to pathogens. Innate immunity provides the initial defense against pathogenic infections, while its propagation leads to the activation of adaptive immune response. At this stage, adaptive immune cells sustain an extremely versatile mechanism of host organism defense in response to the exposure to a known antigen and/or to the reinfection with the same pathogen. In the context of cancer, immunity has clearly emerged as crucial biological event that contributes to the complex process of tumor development [22]. The pathogenesis of ∼ 15–20 % of human tumors is linked to infection-driven inflammation and interestingly, the presence of an inflammatory component characterizes the microenvironment of some tumors that are not causally linked to pathogens [23]. Two general molecular and cellular pathways have been proposed to describe the interaction between inflammation and cancer: the intrinsic pathway and the extrinsic one [23]. The intrinsic pathway which consists in a series of genetic events (e.g. activation of oncogenes, inactivation of tumor suppressor genes, different genetic aberrations) causing neoplastic transformation, initiates the induction of inflammation-related programs which influence the development of an inflammatory microenvironment (e.g. papillary thyroid carcinomas, breast cancers). On the other hand, the extrinsic pathway is driven by inflammatory leukocytes and soluble mediators that sustain inflammatory conditions increasing cancer risk (e.g. colon, prostate, pancreas cancers). All these pathways converge in the first activation of pro-inflammatory transcription factors in cancer cells and in their consequential production of inflammatory mediators (cytokines, chemokines, cyclooxygenase-2, and prostaglandins) [24]. Immune cells of innate system, such as macrophages, myeloid-derived suppressor cells, mast cells, eosinophils and neutrophils, are the first one to be recruited upon inflammation, and their activation contributes to reinforce the pro-inflammatory milieu [25]. The additional secretion of inflammatory mediators leads to the re-induction of the same pro-inflammatory pathways in cancer cells [23]. Independently of the cause that triggers inflammation, these immune signals present in the tumor microenvironment play a crucial role in all stages of cancer evolution, from initiation to metastasis. Indeed, an effective immune response ensues from the harmonious collaboration between innate and adaptive immune system, with the negative feedback of immune checkpoints and immunosuppressive mechanisms. On the other hand, immune response may exert either pro- or anti-tumorigenic effects on tumor microenvironment. A robust body of literature demonstrates that both innate and adaptive immunity are able to execute their role to achieve immunosurveillance, eliminating nascent tumors through the recognition of tumor neo-antigens as non-self [26]. On the contrary, following changes occurring in malignant cell populations or in host immune response, the immune system can fail to eliminate all cancer cells and tumors with reduced immunogenicity may escape the immune attack [27]. This dynamic balance between host-protective and tumor-promoting functions of inflammatory/immune cells have led to hypothesize the concept of cancer immunoediting, the process responsible for both eliminating tumors and modulating the immunogenic phenotypes of tumors that arise in immunocompetent hosts [26]. In conditions of equilibrium, immune system can induce a state of functional dormancy in cells, which can evade from this form of immune-mediated latent tumor through different strategies that allow them to proliferate [26, 28–33]. In established tumors, the escape from immunosurveillance occurs through different mechanisms, either at cancer (e.g. antigen loss, immunogenic tolerance) or immune cell level (e.g. T-cell activation inhibition) [34, 35]. In this second case, the cancer cells immersed in a local immunosuppressed microenvironment are required to mediate the inhibition of effector immune cells, such as T cells, natural killer cells (NK) or dendritic cells and the recruitment of immunosuppressive cells (regulatory T cells and myeloid-derived suppressor cells). Therefore, the heterogeneous composition of tumor microenvironment in terms of immune cell type has the potential to define a pro or an antitumor milieu [36]. Indeed, the presence of tumor-infiltrating lymphocytes (TILs) in human tumors has been described as a prognostic factor, supporting the evidence of immunoediting in this context [37]. Higher frequency of T cells, NK cells and natural killer T (NKT) cells have been correlated with better prognosis in patients with different types of cancer types [38–43]. Specifically, significant association between different subsets of T cells and clinical response have been found in cancer patients. In particular, a positive effect has been ascribed to CD8^+^ T cytotoxic (CTL), CD4^+^ T helper 1 (T_H_1) and T follicular helper (T_FH_) cells, as opposed to the negative role assigned to CD4^+^ regulatory T (T_REG_), T helper 2 (T_H_2) and T helper 17 (T_H_17) cells [44]. In particular, high ratios of CD4^+^/CD8^+^ and T_H_2/T_H_1 lymphocyte markers have been associated with poor prognosis specifically in breast cancer [45]. Among immune cells of myeloid origin, tumor-associated macrophages (TAMs) represent the most abundant cell component of tumor microenvironment, playing an important role in cancer development [46]. Two main different subsets of TAMs have been distinctively described according to their gene expression profiles and pattern of secreted molecules [47]. The natural plasticity of TAMs allows them to easily alter their phenotype during tumor development, converting from a pro-inflammatory (M_1_-like) form at early stages of tumor to a pro-angiogenic/immunosuppressive (M_2_-like) form during later phases of tumor progression (angiogenesis, invasion and metastasis) [48]. Several human tumors are mostly characterized by the presence of TAMs with M_2_-like phenotype [49, 50], while the presence of high levels of either M_2_- or M_1_- like TAMs has been identified as a poor prognostic factor in diverse solid tumors [51, 52]. However, understanding of the exact role of each TAM subset in cancer needs further investigation. Similarly, neutrophils have been demonstrated to exert both antitumor and protumor activities, including the sustainment of T cell responses and the promotion of angiogenesis and metastasis [46]. In addition, myeloid-derived suppressor cells (MDSCs) represent another component of TILs with multiple immunosuppressive functions on both innate and adaptive cells [53]. On the contrary, dendritic cells (DCs) play an important role as antigen presenting cells (APCs), which contribute to mount antitumor CTL immune response [54, 55]. Therefore, the immune-related nature of tumor microenvironment is very complex and depends on finely regulated interactions between cells [56]. This continuous crosstalk between tumor-infiltrating immune cells and cancer cells need to be extensively studied in order to define mechanisms underlying immunosurveillance and tumor immune escape.

## MicroRNAs as regulators of cancer-related immunity in solid tumors

It has been widely demonstrated that differential expression patterns of miRNAs are associated with several human pathologies, including cancer in all its stages [57–59]. To date, miRNAs have been classified either as oncogenic (e.g. miR-155, miR-17-5p or miR-21), or having a tumor suppressor role (e.g. miR-34, miR-15a, let-7) [60–64]. Deregulation of a single miRNA or distinctive miRNA profiles have been correlated with survival, clinical outcome and response to therapy in various solid tumors [65–69]. Interestingly, recent studies have also correlated aberrant expression of crucial proteins related to miRNA biogenesis, with poor outcome [70]. Moreover, miRNAs are important regulators of both innate and adaptive immunity, controlling the maintenance and the development of immune progenitors as well as the differentiation and the functions of mature immune cell (Table 1) [71–74]. Therefore, the complexity of mechanisms underlying the connection between cancer and immunity has led to investigate miRNAs as additional key molecular players. Since specific miRNAs are essential for proper immune cell functioning, it is not surprising that aberrations in expression of immune-related miRNAs can lead to an altered antitumor immune response and contribute to cancer development [75, 76]. Indeed, some miRNAs with validated oncogenic or antitumor properties have shown the potential to exert a modulation of immune cell activity in the tumor microenvironment [20]. A large body of literature has reported different mechanisms by which single immune-related miRNAs have a double role in cancer development and immunity by modulating both immune and non-immune targets (Table 2). In the next subsections we are going to investigate the most relevant miRNAs with immunomodulatory effects, which can function as a bridge between immune response and cancer.

**Table 1:** MicroRNAs involved in Innate and Adaptive Immune System Functions

	Cell lineage	Cellular process	MicroRNAs
*Immune cell progenitors*			
	Hematopoietic stem cells	Cell maintenance	let-7e^a^, miR-29a, miR-99b^a^, **miR-125a**, **miR-126**, miR-212/132 cluster
	Multipotent progenitors	Cell development	**miR-10 family**, **miR-126**, **miR-196b**, miR-221/222
	Common myeloid progenitors	Cell development	**miR-17**, **miR-24**, miR-126, miR-128, **miR-155**, miR-181a
	Common lymphoid progenitors	Cell development	miR-126, miR-128, **miR-146**, miR-181a
	Granulocyte–macrophage progenitors	Cell development	miR-16, miR-103, miR-107
	Macrophage progenitors	Cell development	**miR-17-5p**, **miR-20a**, **miR-106a**
	Granulocyte progenitors	Cell development	**miR-223**
	Erythroid precursors	Cell development	miR-155, **miR-221/222**
	Megakaryocyte precursors	Cell development	**miR-10a**/b, miR-17, miR-20, miR-126
*Innate immunity*			
	Monocytes	Cell differentiation	**miR-17-5p**, **miR-20a**, miR-21, **miR-106a**, miR-155, miR-196b, miR-223, miR-338, miR-342, **miR-424**
		Cell activation	**miR-155**, **miR-424**
	Dendritic cells	Cell differentiation	**miR-21, miR-34a**
		Cell function	miR-10a, miR-148/152**, miR-155**, **miR-223**
	Macrophages	Cell differentiation	miR-15a, miR-16, miR-19a-3p, **miR-21**, miR-107, **miR-146a**, miR-424
		Cell function	**Let-7**, miR-9, **miR-21**, miR-101, miR-**125b**, **miR-146a**, miR-147, **miR-155**, miR-187, miR-212/132 cluster, miR-378, miR-487b, miR-1224
		Cell polarization	let-7c, let-7f, miR-9, miR-21, miR-33, miR-101, miR-124, miR-125, miR-146, miR-147, miR-155, miR-187, miR-223, miR-342, miR-378, miR-511
	Granulocytes	Cell differentiation	miR-15a, miR-21, **miR-27**, miR-196b, **miR-223**
		Cell function	**miR-223**
	Neutrophils	Cell function	**miR-223**
	MDSCs	Cell function	miR-494, miR-17-5p/20a
	Megakaryocytes	Cell differentiation	miR-10a, miR-130a, miR-146a, **miR-150**, **miR-155**, **miR-223**
	Erythrocytes	Cell differentiation	miR-15a, miR-16, miR-24, miR-144, **miR-150**, **miR-155**, miR-221/222 cluster, **miR-223**, **miR-451**
	Natural killer cells	Cell differentiation	**miR-150, miR-181a/b**
		Cell function	miR-15/16, miR-27a, miR-29, miR-30c-1, miR-30e, **miR-155**, **miR-223**, miR-378
*Adaptive immunity*			
	B cells	Cell differentiation	**miR-17/92 cluster**, miR-23a, miR-34a, miR-142, **miR-150**, miR-155, miR-181 family, miR-212/132 cluster
		Cell activation	miR-9, **miR-17/92 cluster**, miR-30, miR-125b, **miR-155**, **miR-181b**, miR-223
	Plasma cells	Cell differentiation	miR-148a
	T cells	Cell differentiation	**miR-17/92 cluster**, miR-21, miR-142-3p, miR-150, **miR-181a**, miR-223
		Cell activation	**miR-155**, miR**-**181a, miR-182, miR-214
	T helper cells	Cell differentiation	miR-125b, **miR-150**
		Cell function	miR-182, miR-214, miR-297, miR-669c
	T helper 1 cells	Cell differentiation	miR-17/92 cluster, miR-29, miR-146a, miR-148a, **miR-155**, **miR-210**, **miR-326**
	T helper 2 cells	Cell differentiation	miR-21, miR-27, miR-28
		Cell function	**miR-155**
	T cytotoxic cells	Cell differentiation	Let-7f, miR-15b, miR-16, miR-17/92 cluster, miR-21, miR-139, miR-142, miR-150, miR-155, miR-342
		Cell function	**miR-17/92 cluster**, **miR-21**, miR-29, miR-23a, miR-24, miR-27a, **miR-30b**, **miR-130/301**, miR-139, **miR-146a**, miR-150, **miR-155**, miR-214
	T regulatory cells	Cell differentiation	miR-17/92 cluster, miR-10, miR-99a/miR-150, **miR-155**
		Cell function	**miR-142-3p, miR-146a, miR-155**
	T helper 17 cells	Cell differentiation	miR-10a, miR-19b, miR-17, **miR-155**, **miR-210**, **miR-212/132 cluster**, miR-301, **miR-326**
	T follicular helper cells	Cell differentiation	**miR-10a**, **miR-17/92 cluster**

The most relevant miRNAs are in bold. MDSCs, Myeloid-Derived Suppressor Cells

^a^Further investigations are required

### MicroRNAs and tumor-associated immune cells

**Fig. 1:**
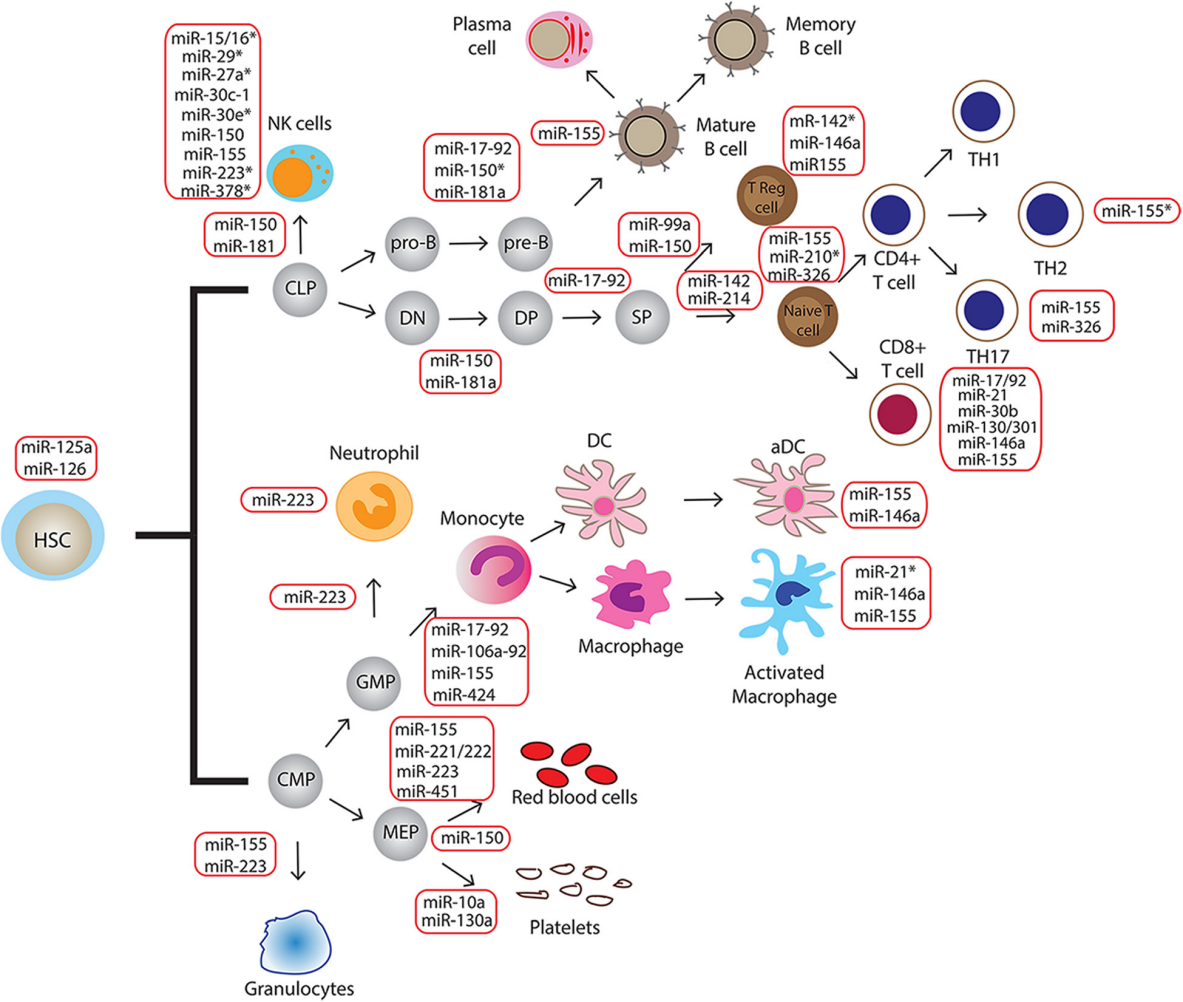
Schematic representation of microRNA regulation in immune cell development and activity. MiRNAs have been demonstrated to function as important regulators of both innate and adaptive immunity, including differentiation and functions of different immune cell subsets. The most relevant miRNAs are included in the figure. The miRNAs in red boxes are involved in regulating the developmental transition indicated by the arrows, as well as the activity of cells in the immune system (full explanation in text). *MicroRNAs involved in negative regulation of immune cell development or function. HSC: Hematopoietic Stem Cell; CMP: Common Myeloid Progenitor; GMP: Granulocyte–Monocyte Progenitor; MEP: Megakaryocyte-Erythrocyte Progenitor; CLP: Common Lymphoid Progenitor; DC: Dendritic cell; aDC: activated Dendritic Cell; DN: Double Negative; DP: Double Positive; SP: Single Positive

The differentiation and the activation of different tumor-associated immune cells have been described to be dependent on expression of specific miRNAs (Fig. 1) [77, 78]. In particular, deregulation of various miRNAs has been identified to affect monocyte-macrophage lineage maturation [79]. Members of miR-17/92 and miR-106a/92 clusters (miR-17-5p, miR-20a, and miR-106a) have been demonstrated to negatively regulate monocyte commitment by targeting Runt-related transcription factor 1 (RUNX1) [80]. Interestingly, modulation of miR-155, miR-125a/b, miR-146a, miR-21, and let-7e have been shown to be crucial for macrophage differentiation and activation into different phenotypes [81]. Specifically, the upregulation of these miRNAs have been observed to occur in macrophages after activation of Toll-like receptor (TLR) signaling and to sustain (i.e. miR-155, miR-125a/b) or repress (i.e. miR-146a, let-7e) pro-inflammatory M_1_- like TAM activation [79]. In the context of macrophage cell functions, miR-146a and miR-155 are also the two most well characterized miRNAs regulating immune response mediated by these cells [76]. These two miRNAs act as mediators of inflammatory stimuli with opposite effects on inflammatory response, miR-146a as negative and miR-155 as positive regulators of immune response through the direct targeting of IRAK1 and TRAF6, and SOCS1 and BCL6, respectively [82, 83]. However, this gene network is a part of a bidirectional mechanism in which inflammatory pathways are able to modulate miR-146a and miR-155 expression, and in turn innate immune response [83–86]. Along with TAMs, NK cells are another key component of the innate immune response and their differentiation has reported to be determined by miR-150 and miR-181a/b expression level [87]. In addition to tumor-associated innate immune cells, miRNAs have been found to regulate cell differentiation and functions of different T cell subsets (Fig. 1). Specifically, T helper effector differentiation has been found to be regulated by miR-155 expression in favor of T_H_1 phenotype, by miR-326 in promoting T_h_17 differentiation, and by miR-10a and miR-17-92 cluster regulating T follicular helper maturation [88–90]. In the lineage of CTL cells, the maturation and the activation of cells into effector or memory cell subsets have been demonstrated to be promoted by different miRNAs, including miR-17/92, miR-21, miR-30b and miR-155 [91]. On the contrary, miR-130/301 and miR-146a have displayed inhibiting effects on CTL immune responses [91]. miRNA roles have been also elucidated in T_reg_ cell biology, with particular attention to miR-142-3p, miR-146a and miR-155 for cell function [92, 93].

All these infiltrating inflammatory cells are recruited to the tumor microenvironment and their activation can be modulated by molecular signals produced by stromal and malignant cells [94]. In this context, distinctive miRNA-mediated mechanisms have been identified in different models of cancer (Table 2). In breast cancer miR-19a-3p has been reported to regulate the switch of TAMs from a M_2_-like phenotype into M_1_-like macrophages by targeting the *Fra-1* proto-oncogene and other genes of its downstream signaling pathway (VEGF, STAT3 and pSTAT3), and to contribute to the inhibition of metastasis development [95]. In particular, *Fra-1* has been already demonstrated to have a key role in the polarization of TAMs from the M_1_- to the M_2_-like phenotype [96]. Specifically, in the Balb/c mouse model, in vivo miR-19a-3p intratumoral injection has been found to both decrease the population of M_2_-like TAMs and inhibit lung metastasis of 4 T1 breast cancer cell-derived tumors [95]. Similarly, the miR-23a/27a/24-2 cluster has been demonstrated to mediate macrophage polarization and to contribute to tumor progression in breast cancer [97]. These studies support the concept that the modulation of the expression of single miRNAs (miR-19a-3p or miR-23a/27a/24-2 cluster downregulation) can promote the activation of specific signaling pathways, and the differentiation of a specific immune cell type (M_2_ phenotype of TAMs) in the tumor microenvironment.

**Table 2:** Main deregulated microRNAs/targets, and the biological roles in immune- and cancer-related pathways in solid tumors

Cancer type	miRNA	Expression status and cell localization^b^	Target	Immune-related role	Cancer-related role	Ref^c^
Breast	↑miR-10b	Cancer cells	↓MICB	Suppression of NK-mediated killing of tumor cells	Metastasis development^d^	[107]
	↑miR-19a-3p	M_2_ Macrophages	↓FRA-1	Macrophage polarization	Inhibition of cancer progression and metastasis development	[95]
	↑miR-21	Cancer cells	↓PIAS3	Reduced chemokine production and lymphocyte migration, immunoresistance to cancer immunotherapy^d^	Cancer cell survival, cell proliferation	[137]
	↓miR-23a/ 27a/24-2	Macrophages	↑A20 ↑JAK1 ↑STAT6	M_2_ Macrophage polarization	Xenograft tumor growth	[97]
	↓miR-126/126^a^	Cancer cells	↑SDF-1α	Downregulation of Ccl2 expression, Suppression of Inflammatory monocyte recruitment	Repression of MSC recruitment, lung metastasis promotion	[100]
	↓miR-146a	Cancer cells	↑IRAK1 ↑TRAF6	Modulation of inflammation^c^	Cell Invasion and Migration impairment (NF-kB signaling block)	[92]
	↑miR-155	Cancer cells	↓SOCS1	STAT3 signaling activation	Cancer cell proliferation, colony formation, and xenograft tumor growth	[141]
		Myeloid cells	↓SHIP1	Tumor-infiltrating innate immune cell recruitment	Antitumor activity	[98]
	↑miR-223^a^	M_2_ macrophages and cancer cells	↓MEF2C^d^	Macrophage differentiation^d^	Promotion of cancer cell invasion	[163]
	↑miR-494	MDSCs	↓PTEN	Accumulation of MDSCs	Tumor cell invasion and metastasis development	[117]
Gastric	↓miR-146a	Cancer cells	↑IRAK1 ↑TRAF6 ↓IL8	Modulation of inflammation^d^	Antitumor activity	[146]
Ovarian	↑miR-20a	Cancer cells	↓MICA/B	Suppression of NK-mediated killing of tumor cells	Long-term cellular proliferation, invasion capabilities	[104]
	↓miR-199a	Cancer cells	↑IKKβ	Cytokine production	Tumor progression, chemosensitivity (NF-kB signaling modulation)	[148]
	↑miR-424	Cancer cells	↓PDL1 ↓CD80	T cell activation	Chemosensitivity	[151]
Colorectal	↓miR-17-5p/miR-20a/miR-124	MDSCs	↑STAT3	Inhibition of immunosuppressive potential of MDSCs	Tumor growth	[142–144]
	↑miR-21/miR-29b^a^	Cancer cells and immune cells	↑IL-6 (Indirectly)	Activation of pro inflammatory immune cells^d^	Promotion of cancer cell invasion, tumor progression^d^	[164]
Hepatocellular	↑miR-20a, miR-96, miR-106b	Cancer cells	↓MICA	Suppression of NK-mediated killing of tumor cells	Long-term cellular proliferation, invasion capabilities^d^	[108]
HBV^+^/Hepatocellular	↓miR-34a	Cancer cells	↑CCL2	Regulation of Treg recruitment	Suppression of tumor growth/metastasis development	[113]
Melanoma	↓miR-34a/c	Cancer cells	↑ULPB2	Suppression of NK-mediated killing of tumor cells	Cell cycle arrest, senescence, apoptosis	[111–112]
	↓miR-17	T cells	↑STAT3	Impairment of T cell response^d^	Tumor growth^d^	[142]
Melanoma and Lewis lung cancer	↓miR-155	Immune cells	↑HIF1a	Recruitment of MDSC cells to tumor microenvironment	Promotion of tumor growth^d^	[118]
Lung	↑miR-23a	T cells	↓BLIMP1	Suppression of CD8^+^ T cell function^d^	Tumor progression, TGF-β-mediated immune evasion^d^	[145]
Glioma	↓miR-124	T cells	↑STAT3	Impairment of T cell responses	Tumor growth	[143]
Various solid tumors	↓miR-29	Cancer cells	↑B7-H3	Inhibition of NK and T cell function^d^	Protumor activity^d^	[114–115]
	↓miR-214^a^	Cancer cells and CD4+CD25+ T cells	↓PTEN	Expansion of Treg cells	Promotion of tumor growth	[166]

^a^ Detailed mechanism involving microvesicles-cell interactions (see also subsection “MicroRNAs and cell-to-cell communication”)

^b^ These data are referred to studies in either tissue samples or in vitro/in vivo models

^c^ Reference number listed in bibliography

^d^ Further investigation is needed

Upregulation of miRNA or miRNA target. Downregulation of miRNA or miRNA target

HBV, Hepatitis B Virus; MDSCs, Myeloid-Derived Suppressor Cells; NKs, Natural Killer cells; TAMs, Tumor Associated Macrophages

Interestingly, miR-155 has been also reported to mediate the antitumor potential of distinctive immune cell subsets in breast cancer. In particular, miR-155 upregulation has been recently demonstrated to be required in the myeloid cell compartment for the promotion of antitumor immunity in early stages of breast cancer carcinogenesis [98]. In a spontaneous breast cancer model, specific miR-155 knock down in myeloid cells is able to induce faster tumor growth, reduction of M_1_-like TAMs and enrichment of protumor cytokines within tumor milieu, all concurring to create an immunosuppressive microenvironment [98]. In particular, the proposed mechanism involves the regulation of SHIP1, which is the main negative regulator of the pro-inflammatory PI3K/AKT pathway. The inhibition of this pathway was demonstrated to revert the common pro-inflammatory and protumor events mediated by AKT activation [99].

In the same direction, miR-126/126* pair has been shown to have an antitumor role by inhibiting breast cancer cell invasion and metastasis [100], either through the direct targeting of stromal cell-derived factor-1 alpha, SDF-1α, and with the indirect suppression of chemokine (C-C motif) ligand 2, CCL2, in cancer cells. These two chemokines mediate the sequential recruitment of two different non malignant cell types to primary tumor site: SDF-1α is responsible for attraction of mesenchymal stem cells (MSCs), while the second for inflammatory monocytes. MSCs are supposed to create a paracrine loop with cancer cells to induce cell invasion and migration, meanwhile monocytes act to promote the extravasation of tumor cells [101, 102]. Therefore, miR-126/126* pair is able to modulate the composition of the microenvironment of primary tumors in order to contrast breast cancer metastasis. These findings are perfectly in line with discoveries correlating reduced expression of miR-126 to poor metastasis-free survival of breast cancer patients [103].

As previously described, the complexity of tumor microenvironment includes innate immune components recruited to eradicate latent cancer cells. Among them, NK cells are a subset of lymphocytes that can rapidly respond to the presence of tumor cells and initiate an antitumor immune response. NK cells express receptors through which they are capable to detect their targets on cancer cells. MiR-20 has been demonstrated to regulate NK cytotoxicity in ovarian cancer through the targeting of MICA/B, a MHC class I chain-related molecules widely expressed on epithelial tumor cells [104]. This protein is recognized by NK cells through the NK group 2 member D receptor (NKG2D), whose pathway is critical for direct recognition of malignant cells by immune surveillance system [105]. In vitro and in vivo studies have shown that miR-20-mediated downregulation of MICA/B induced the reduction of NKG2D recognition resulting in the diminished killing of malignant cells by NK compartment, thus leading to enhanced tumor cell survival in vivo [106]. The same mechanism has been demonstrated for miR-10b/MICB pair in murine breast cancer model, and for miR-20a, miR-93, miR-106b/MICB pair in hepatocellular cell lines [107, 108]. These data propose a miRNA-based immune escape mechanism for tumor cells, which can partially explain the correlation between overexpression of these miRNAs and poor prognosis in cancer patients.

Similarly to human ovarian cancer cells, human melanoma cells have been reported to express NKG2D receptor ligands such as MICA and ULBP2 [109]. Recently, Heinemann A. et al. have identified serum ULBP2 overexpression as a strong independent predictor of poor prognosis in melanoma patients [110]. The same group also demonstrated that the tumor suppressor miR-34a/c repressed ULBP2 expression by directly binding to the 3’-UTR [111]. Together with the fact that miR-34a/c expression is frequently lost in cancer, these miRNAs might be crucial for tumor immune surveillance [112]. This immuno-suppressive mechanism is also suggested to predispose HBV-Positive hepatocellular carcinoma (HBV-HCC) patients to the development of intrahepatic venous metastasis. Specifically, miR-34a deregulation has been linked to immune escape mechanism in HBV-HCC, whose development is supposed to be associated with HBV and HCV virus infection [113]. In particular, TGF-β/miR-34a/CCL22 axis induced the recruitment of T_reg_ cells that are known to have an inhibitory role in the immune system and, ultimately, to participate to escape immune surveillance helping tumor cells [113]. Interestingly, TGF-β signaling activation induces the suppression of miR-34a, CCL22 expression and in turn recruitment of T_reg_ cells in liver microenvironment.

In the context of immune cell function regulation, miR-29 has been identified as a negative regulator of B7-H3 protein, which is a surface immunomodulatory glycoprotein inhibiting NK and T cell functions [114]. Specifically, mir-29 and B7-H3 expression levels have been found inversely correlated in both solid tumors and cancer cell line experiments [115]. The downregulation of miR-29 family members has been reported in many cancers, where they influence cell proliferation, apoptosis and metastasis development through the modulation of different targets [116]. Additional in vivo and in vitro studies have to be performed in order to validate the role of miR-29 in the promotion of antitumor immunity mediated by NK and T cell. Along with NK and T cells, the expansion and function of MDSCs depend on soluble factors released by tumor and stromal compartments and by activated immune cells [116]. Recently, it has been demonstrated that two different miRNAs, miR-494 and miR-155, are fundamental for the recruitment of MDSCs to the tumor site, contributing to the modulation of their immunosuppressive function and to tumor growth in breast cancer and glioma models, respectively [117, 118].

### MicroRNAs and cancer-related immune pathways in solid tumors

Cancer and immune cells produce various growth or angiogenic factors, proteinases, chemokines and cytokines, which contribute to removal of tumor cell or to the formation of an immunosuppressive microenvironment [119, 120]. In particular, the activation of specific transcription factors and the presence of primary inflammatory cytokines represent key connection elements between immune and cancer cells [121, 122]. Among them, nuclear factor kappa-B (NF-kB), transducers activator of transcription 3 (STAT3), tumor necrosis factor (TNF) and transforming growth factor β (TGF-β), have been demonstrated to mediate the activation of numerous oncogenic pathways [122–124]. NF-kB is a major regulator of inflammation and innate immunity, and its aberrant regulation has been described in many human tumors [124]. Different upstream stimuli have been identified to trigger NF-kB pathway, including pro-inflammatory cytokines (e.g. TNF-α, IL-1β) and microorganism infections (bacterial and viral infections), which both determine the activation of the IkB kinase (IKK) complex. This protein unit phosphorylates inhibitor of NF-kB and allows the translocation of NF-kB into nucleus [125]. The downstream effects of NF-kB are cell-type dependent, consisting in induction of gene expression related to pro-inflammatory signals in inflammatory cells and of anti-apoptotic genes in tumor cells, favoring tumor development [125]. One of the most important activators of NF-kB signaling is TNF-α, which binds to its specific receptor expressed by immune or cancer cells [126]. This pro-inflammatory cytokine has been also demonstrated to exert tumor–promoting activities, including promotion of angiogenesis and metastasis [127]. Similar to NF-kB, STAT3 is constitutively activated in both tumor and immune cells increasing tumor cell proliferation, survival and invasion and is activated by NFkB-induced genes such as Interleukin 6 (IL6) [128–129]. On the contrary, STAT3 signaling is required for immunosuppressive and pro-tumorigenic functions of immune cells of both innate (MDSCs, TAMs) and adaptive system (T_reg_, T_h_17), opposing the role of NF-kB in favoring antitumor immune response [130, 131]. Immunosuppressive and anti-inflammatory signals derive also from the action of TGF-β, which attenuates the production of pro-inflammatory cytokines [132]. Furthermore, TGF-β is known to be a key cytokine during carcinogenesis, being secreted and upregulated in a wide range of tumors [133–136]. Therefore, different signaling represent crucial pathways in the model of origin of cancer-related inflammation and large body of literature supports the central role of miRNAs as both down- or up-stream modulators of the activation of these factors (Table 2).

Among these miRNAs, miR-21 and miR-155 have been found to be part of the complex immune regulatory network in the context of breast cancer [137–141]. These two oncomiRNAs are linked in different ways to the constitutive activation of STAT3 pathway, promoting the expression of immunosuppressive factors and thus having immune suppressive effects. Inhibition of miR-21 expression levels in MCF7 cell line has recently been reported to increased chemokine and lymphocyte migration, which is paralleled to an increase in levels of PIAS3, an inhibitor of activated STAT3, and of STAT3 phosphorylation, making miR-21 a good therapeutic target [137]. In particular, this mechanism is explained by the activity of miR-21, which inhibits the release of RANTES and IP-10, T cell chemoattractants, through the targeting of PIAS3 [137]. These data have demonstrated a positive relationship between miR-21 and STAT3 in a tumor cell line context, as previously reported [138, 139]. In addition to the protumor activities affecting cell survival and proliferation, miR-21/STAT3 network could contribute to create an immune suppressive tumor milieu, thus supporting the miRNA role as regulator of drug resistance [140]. Similarly to this miRNA, miR-155 exerts its oncogenic role by negatively regulating the tumor suppressor gene of cytokine signaling 1 (*Socs1*) and consequently promoting cell proliferation, colony formation and xenograft tumor growth in breast cancer model [141]. The immunomodulating property is represented by the constitutive activation of STAT3 signaling as tumor promoting inflammatory mechanism.

Other miRNAs have been linked to the regulation of STAT3 signaling. As previously described, effective adaptive immune responses have a key role in contrasting tumor progression and different immune cell subsets contribute to maintain this potential. In this context, STAT3 mRNA has been demonstrated to be directly targeted by miR-17-5p, miR-20a and miR-124 in different cancer models where forced miRNA upregulation has been showed to sustain T cell response in favor of an antitumor activity [142–144]. Conversely, miR-23a contributes in suppressing CTL function, at least in a murine model of lung cancer [145]. Specifically, the TGF-β-mediated upregulation of miR-23a in CTL cells induced a tumor immune-evasion mechanism by targeting the transcription factor BLIMP-1, in turn promoting tumor progression [145].

As previously mentioned, miR-146a is a miRNA involved in the control of both inflammatory response to infection and of innate immune system. In particular it has been described as a NF-kB-dependent gene, which in turn downregulates the expression of immune target genes, such as IRAK1 and TRAF6 (two adaptor molecules downstream of toll-like and cytokine receptors), and represses NF-kB signaling in LPS-stimulated monocytes [83]. MiR-146a axis has been investigated also in gastric cancer cells, where a link between miR-146a and the inflammatory response to Helicobacter pylori has been envisaged [146]. Similarly, forced ectopic expression of miR-146a in MDA-MB-231 breast cancer cell line inhibits endogenous NF-kB expression and activity through IRAK1 and TRAF6 targeting, reducing their metastatic potential [147]. Further investigations are needed in order to fully elucidate how miR-146a contribute to tumor development.

A study by Chen R. et al. has described a similar miR-199a/IKKβ/NF-kB axis also in epithelial ovarian cancer cells (EOCs) [148]. MiR-199a has been identified as a negative regulator of IKKβ mRNA, which encodes for the β subunit of IKK, the direct upstream activator of NF-kB pathway [149]. In EOCs, IKKβ contributes to a pro-inflammatory environment by functionally inducing NF-kB pathway through the activation of Toll-like receptor 4 (TLR4)-MyD88 signaling sustaining tissue repair processes and the secretion of pro-inflammatory signals [150]. Consequently, EOC cells are induced to secrete pro-inflammatory/protumor cytokines, including IL-6, IL-8, MCP-1, MIP-1α, RANTES, GRO-α, GM-CSF and MIF. MiR-199a targets IKKβ resulting in the inhibition of NF-kB signaling. Therefore, the loss of this anti-inflammatory miRNA may be an important step that contributes to tumor progression. These data may have important implications in tissue repair, tumor progression and chemoresistance. Interestingly, chemosensitivity of EOCs has been shown to be restored by miR-424 overexpression, which activates T cell immune response through direct targeting of critical immune checkpoints, such as the programmed death-ligand 1 (PD-L1) and CD80 [151].

### MicroRNAs and cell-to-cell communication

**Fig. 2:**
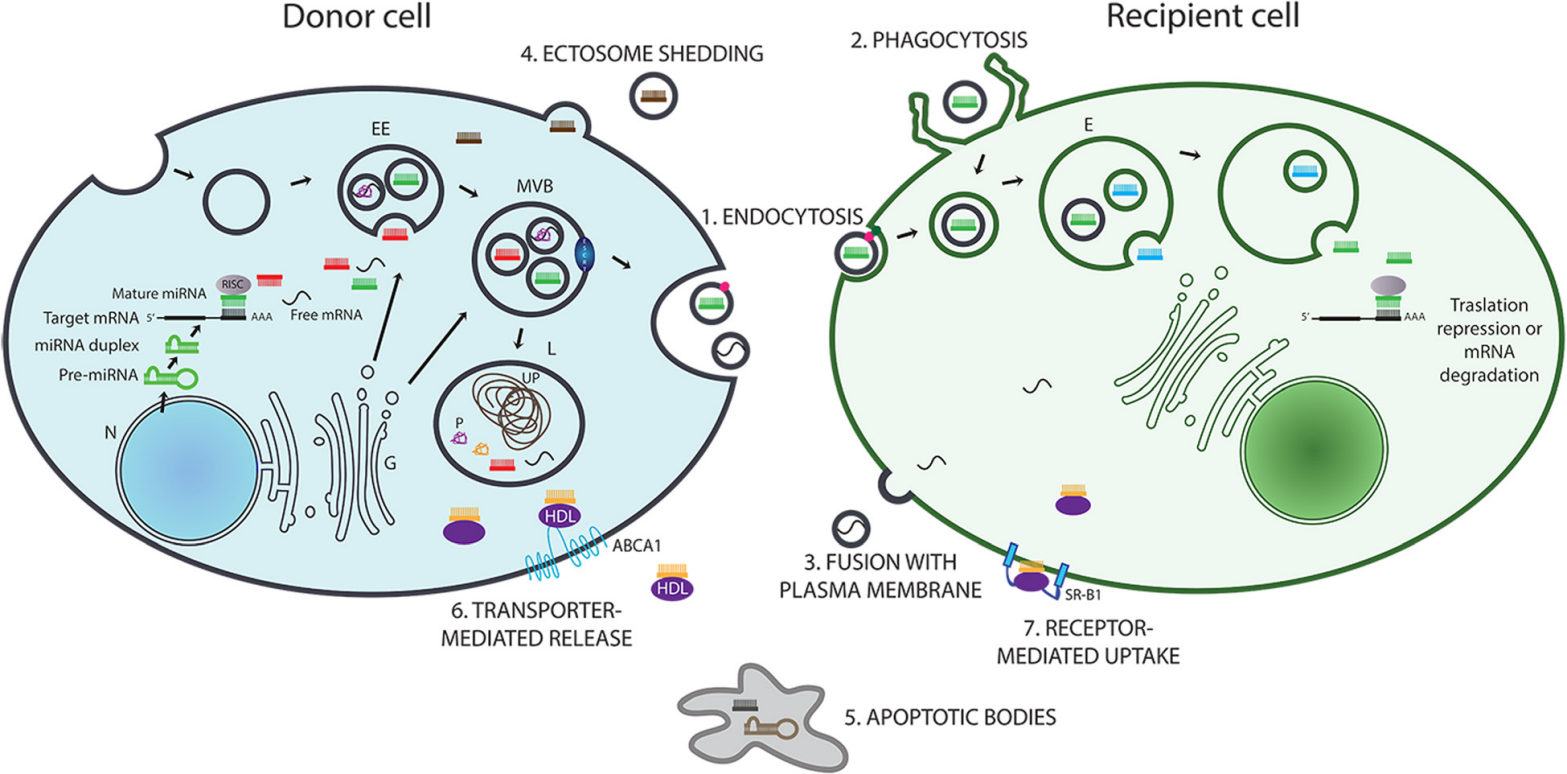
Mechanisms of RNA transfer in cell-to-cell communication. Mechanisms underlying the transfer of RNA molecules between cells are mainly based on two systems, vesicle- and protein-mediated transport. (1,2,3) After exosome release from donor cell, RNA content is delivered into recipient cell by (1) the fusion of the exosome with the recipient cell membrane, by (2) phagocytosis- or (3) endocytosis-like internalization of the exosome. RNA molecules can be exported and transported out of the cells by microvesicles also as (4) shedding ectosomes or (5) apoptotic bodies. (6,7) Different protein complexes (violet boxes) including Argonaute, NPM1 and HDL proteins, bind miRNAs and are transferred out of the cell through (6) transporter-mediated release (ABCA1) and are translocated to target cell by (7) receptor-mediated uptake (SR-B1). All these pathways result in the delivery of microRNA or mRNA molecules to the cytosol of the recipient cell where they may contribute to post translational gene regulation. E: Endosome; EE: Early Endosome; G: Golgi; L: Lysosome; MVB: Multivesicular Bodies; N: Nucleus; P: Protein; UP: Undigested Protein

miRNAs have emerged as crucial mediators of intercellular communication occurring between immune and tumor cells within the tumor microenvironment [152]. The vast majority of studies described the mechanisms of deregulation of endogenous miRNAs in immune or tumor cells, which can modulate the cancer-related immune response, thus also affecting tumor progression. More recently, a mechanism of indirect cell-to-cell communication has been described, showing that exogenous miRNAs can be transferred from a donor to a recipient cell in order to modulate gene expression [21]. This process is based on cell-derived extracellular vesicles (EVs) containing both proteins and RNAs, including miRNAs and mRNAs. Generally EVs are subdivided into three major classes of particles according to size, ectosomes or shedding vesicles (200–1000 nm) and exosomes (30–200 nm), which differ from apoptotic bodies (0.5–3 μm) that derive from cells in apoptosis or under stress [153]. In particular, exosomes have been identified as the most important carriers of functional miRNAs [21]. Exosomes are small membrane-derived vesicles of endocytic origin, which can be released by different types of cell, including immune and tumor cells, under both normal and pathological conditions [154]. The mechanism of horizontal exchange of exosome containing miRNAs between cells is described in Fig. 2. Interestingly, the composition of RNA molecules in exosomes is different from the content of cell of origin suggesting the existence of not well defined active mechanisms for sorting specific RNAs into exosomes [155–157]. In particular, the ESCRT (endosomal sorting complex required for transport) protein complex is likely to be involved in some of these processes [157–159]. However, the exact nature of these mechanisms remain to be extensively characterized. The transfer of this genetic information can alter gene expression in neighboring and even distant cells. Recent data have demonstrated involvement of exosome at different levels, including immune responses and cancer [160–162]. These findings suggested that exosome-mediated miRNA transfer between immune and tumor cells could be crucial to identify new miRNAs as modulators of tumor microenvironment and potential target for cancer immunotherapy. Several recent studies provided evidences of this hypothesis (Table 2). A study by Yang M. et al. firstly demonstrated that after transfection into IL-4 activated M2 macrophages, the exogenous miR-223 can shuttle into co-cultivated breast cancer cells from M2-derived exosomes containing miRNA. Interestingly, exosome–transferred miR-223 stimulates the invasive behavior of breast cancer cells by targeting of Mef2c/β-catenin pathway, thus leading to increase cell migration [163]. A more complex regulatory loop between cancer and immune cells has been described in in vitro co-culture model of colorectal cancer: the secretion of IL-6 from immune cells promotes invasiveness of cancer cells, which in turn induces immune-related IL-6 production and miR-21 release through the tumor-derived secretion of miR-21 and miR-29b in the tumor microenvironment [164]. As result, this mechanism mediated by exogenous miRNAs is suggested to be able to support the maintenance of a pro-tumorigenic inflammatory environment. Moreover, the two tumor-secreted miR-21 and miR-29b have been reported to act as ligands binding to receptors of TLR family of macrophages, producing pro-inflammatory signals in the tumor microenvironment and pro-metastatic potential in in vivo models [165]. Similarly, miR-214 has been identified as another tumor-secreted miRNA capable to support simultaneously host immune suppression and tumor growth in in vivo mice models [166]. Specifically, tumor-derived miR-214 was delivered into peripheral CD4^+^ T cells and found to induce T_reg_ expansion by targeting phosphatase and tensin homolog (PTEN) protein. The secretion of higher levels of IL-10 by miR-214-induced T_reg_ has been demonstrated to promote tumor growth in nude mice [167]. The dual role of tumor-derived exosomes in the modulation of immune responses and in the mediation of tumor progression has been also investigated at circulating level in cancer patients [167–170]. In particular, higher level of circulating exosomes have been found in breast or ovarian cancer patients compared to healthy individuals, and serum exosomes derived from patients with oral or ovarian cancer have been characterized as inhibitors of T cell functions [167–170]. In this context, interestingly circulating tumor-derived exosomes isolated from serum of nasopharyngeal carcinoma patients have been characterized as miRNA-enriched vesicles able to impair in vitro T cell function interfering with ERK and STAT signaling pathways in T cells [171]. In addition to the exosome mediation, miRNA transfer has been demonstrated to happen directly via intercellular contact through gap junctions. Specifically, human macrophages have been reported to be able to secrete miR-142 and miR-223, which are transferred into hepatocarcinoma cells (HCCs) [172]. These miRNAs affect posttranscriptional regulation of target proteins in HCC cells, in particular inducing decreased expression levels of stathmin-1 (STMN1) and insulin-like growth factor-1 receptor (IGF-R1), and, importantly, inhibiting proliferation of cancerous cells in in vitro experiments [172]. In opposition to its antitumor role in HCC model, M2-derived miR-223 has been previously shown to stimulate proliferation in breast cancer cells because of the targeting of Mef2c/β-catenin pathway related to the inhibition of cell migration [163, 173, 174].

More data regarding different mechanisms of miRNA transfer from non-immune stromal to tumor cells have been published [175]. All these findings highlight the potential role of shuttled miRNAs in mechanisms used by cancer cells to evade immunosurveillance and to sustain tumor progression.

## MicroRNA-based immune response as potential target for anticancer immunotherapies

**Fig. 3:**
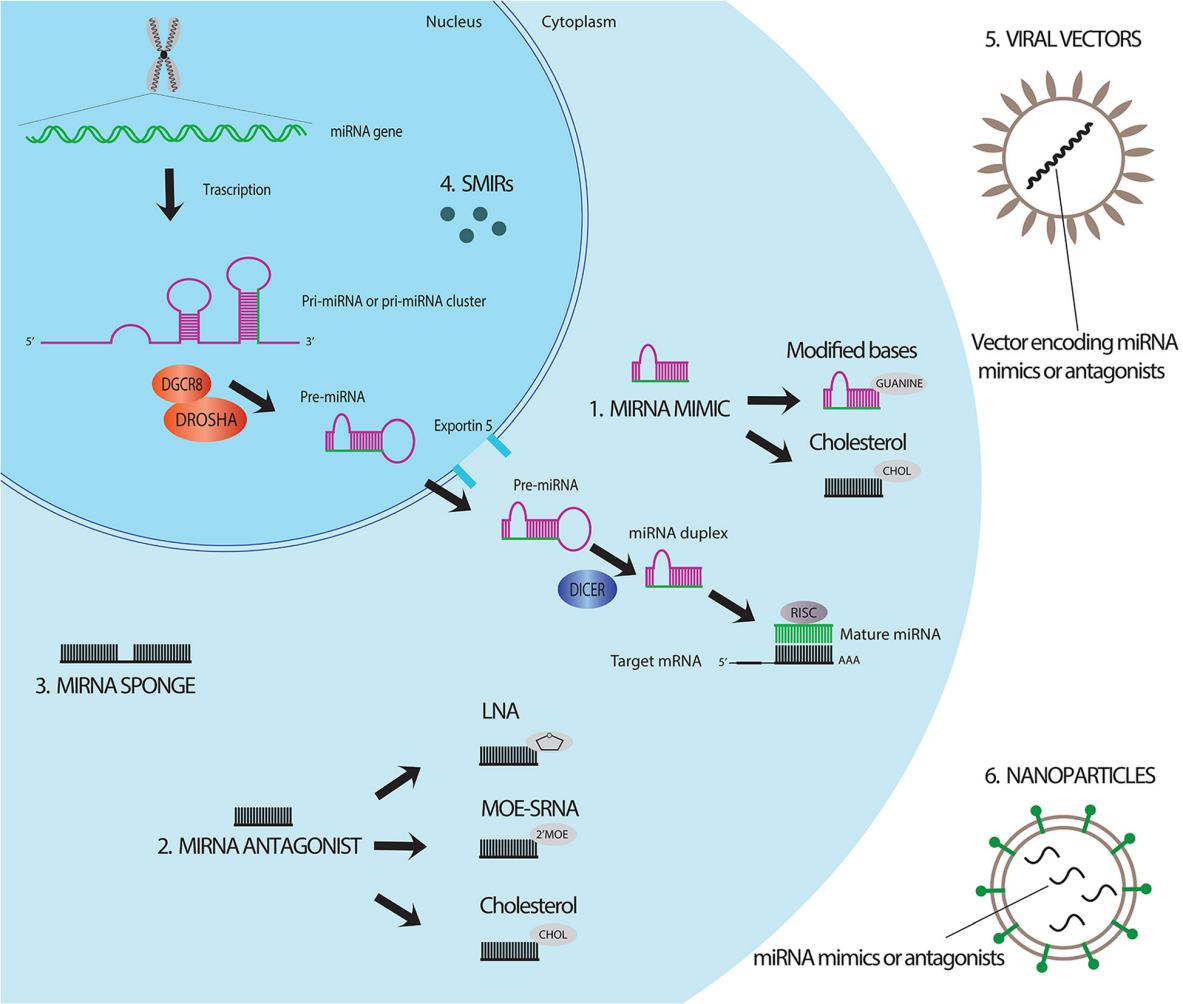
MicroRNA-based strategies for anti-cancer therapy. The main strategies for the modulation of miRNA activity are basically based on enhancing or inhibiting the expression of specific miRNAs with miRNA mimics (1) or miRNA antagonists (2), respectively. Modified miRNA molecules have been developed to increase the stability of miRNA mimics and miRNA antagonists, including miRNA mimics containing modified cyclopentyl-guanine based, cholesterol-conjugated 2′-O methyl-modified miRNA mimics/anti-miRs, locked nucleic acid (LNA)-modified anti-miRs and 2′-O-methoxyethyll-4′-thioRNA (MOE-SRNA). A different approach consists in miRNA sponges (3), which are complex constructs able to interfere with miRNA/mRNA interaction. Interference at miRNA biogenesis level is obtained with small-molecule inhibitors of miRNAs, SMIRs (4). Modified or unmodified miRNA modulators can be delivered to target cells by using viral (5) or non-viral vectors consisting in different types of biocompatible and biodegradable nanoparticles (6)

The potential of miRNAs in regulating different cellular pathways and as mediators of interactions between cells make them ideal drug targets. Indeed, different local and systemic delivery strategies are already under investigation [176]. Their ultimate aim is either enhancing or inhibiting the expression of specific miRNAs acting as tumor suppressor genes or oncogenes, respectively [176]. These effects can be achieved by targeting miRNAs at different levels of their biogenesis and activity (Fig. 3). In the context of cancer cell targeting, promising results have been obtained with the use of miRNA antagonists and miRNA mimics in preclinical studies [177, 178]. miRNA antagonists are single-stranded oligonucleotides complementary to miRNA sequences, designed to target and functionally reduce miRNA activity [179]. On the other hand, restoration of tumor suppressor miRNA level is obtained by the delivery of chemically synthesized short double-stranded oligonucleotides, which functionally mimic pre-miRNA duplexes [180]. To date, these two strategies are under investigation in clinical trials based on targeting of miR-122 and miR-34a for the treatment of hepatitis C virus and advanced HCC, respectively [181]. Due to the recent advances in the understanding of specific miRNA modulatory mechanisms that influence the immune system and tumor-mediated immunity, researchers are now developing novel miRNA-based interventions for cancer immunotherapy. Currently, different immunotherapies have been approved aimed to activate antitumor immunity, including passive immunization with monoclonal antibodies, systemic delivery of cytokines and addition of immune adjuvants into the tumor microenvironment [182]. Among them, the use of tumor targeting monoclonal antibodies seems to be the most promising approach for some hematologic and solid tumors [183]. Novel antibody-based approaches are under development in order to block immunosuppressive networks or stimulate antitumor cytotoxicity [184–188]. In this context, the regulation of cancer-related immune responses through the fine tuning of immune-related miRNA expression could contribute to enhance antitumor immunity and at the same time inhibit tumor development. Interestingly, miRNAs can be targeted not only in cancer cells but also in stromal cells, such as tumor-associated fibroblasts and lymphocytes, which are essential for tumor formation, progression and metastasis. In this case, successful delivery directed to specific tissue and cell, represents a big challenge for in vivo miRNA-mediated immunomodulation. Different types of vehicles have been synthesized as biodegradable and biocompatible carriers of miRNA mimics and miRNA antagonists, including liposomes, polymers, nanoparticles and viral agents [182]. The versatility of liposomal carriers have made them suitable elements for designing of co-delivery system of miRNAs and small-molecule drugs which concurrently are able to target the same cancer cell resulting in an effective synergic antitumor effect. Firstly employed for small conventional drugs and siRNA delivery in clinical trials, liposomal formulation of miR-34a mimic is currently used in a Phase I clinical trial of patients with advanced HCC [17]. Polymeric micelles, polymeric nanoparticles and carbon nanomaterials have already been employed in co-delivery system of different miRNAs and chemotherapeutics in different tumor models [189–192]. Interestingly, systematic intensive infiltration of myeloid leukocytes in solid tumors and their enhanced endocytic activity make these cells the ideal targets for nanocomplex-mediated delivery. Accordingly, a recent work has obtained the induction of miR-155 activity selectively in DCs in ovarian cancer microenvironment using a non-viral approach, resulting in the promotion of T-cell mediated protective immunity and therefore antitumor responses [193]. In the same context, downregulation of miR-31/miR-214 and upregulation of miR-155 are capable to selectively reprogram normal human fibroblasts into tumor-promoting cancer-associated fibroblasts (CAFs) [194]. Furthermore, downregulation of miR-214 has also been demonstrated to directly influence chemokine (C-C motif) ligand 5 CCL5 production, whose increased levels lead to enhanced tumor growth, by stimulating OvCa cells invasion [195]. Therefore, miR-31/miR-214 restoration and inhibition of miR-155 in protumor CAFs could be considered as potential immunotherapeutic options for ovarian cancer. Similarly, miR-21 has been identified as a potential immunotherapeutic target for its ability to positively regulate STAT3 signaling, which is a prerequisite for effective T cell therapy. In addition, miR-21 can suppress T cell priming and impair responses triggered by anti-mycobacterial vaccination [196].

Opposed to previous described non-viral delivery approach, viral vectors have been investigated as potential carriers of miRNAs in order to improve the efficiency and the specificity of the systemic delivery. Different examples of application of virus can be mentioned, starting from lentiviral vectors containing miR-494 antagonists with the potential of reducing tumor-infiltrating MDSCs and their protumor activity in in vivo breast cancer model [117]. Lentiviruses are able to integrate their reversed DNA into human cells, implicating the potential risk of different oncogenic pathways’ activation. Adenovirus and adeno-associated virus are more suitable for therapeutic purposes, due to their non-integrative activity [197]. However, the general increased immunogenic microenvironment and the limits in their large-scale production make viral vectors a less safe delivery system than non-viral approach.

In the context of extracellular vesicle-based therapy, the identification of exosomes as miRNA carriers and mediators of communication between cells of tumor microenvironment constitutes an opportunity to study new targeted approaches [198]. Similar applications have been already investigated using tumor-derived EVs to deliver a therapeutic miRNA to breast cancer cells with specific phenotypes [199]. In particular, the tumor suppressor miRNA let-7a has been efficiently delivered to epidermal growth factor receptor (EGFR)-expressing breast cancer cell, exerting the in vivo inhibition of tumor development. The specific targeting of receptor-expressing cells was obtained by using exosomes derived from engineered donor cells with EGFR ligand on plasma membrane. As regards the immune system, EVs derived from immune and non-immune cells are able to positively and negatively regulate the immune response [200]. Thus, cell-derived exosomes containing immune-related miRNAs could have the potential to be used as therapeutic agents to enhance immunostimulatory signaling pathways and antitumor immunity. In this context, tumor-derived exosomes has been primarily investigated, showing their potential to concur to immune evasion [201, 202]. Recently, miRNAs have been reported to be associated with the RISC-Loading Complex in breast cancer-derived exosomes [203]. These entities display cell-independent miRNA biogenesis able to sustain Dicer-dependent mRNA targeting in recipient cells. Specifically, cancer exosomes can induce malignant transformation of normal epithelial cells in a Dicer-dependent manner. This work contributes to propose miRNA-containing exosomes as active players of cancer progression. However, the use of exosomes as carriers of miRNAs in cancer therapies is only at the beginning and needs to be further investigated.

## Conclusions

The importance of specific miRNAs for immune cell development and function has been widely demonstrated, and their association with different human diseases is a matter of fact. Alteration in levels of these immune-related miRNAs could determine the modulation of immune pathways and the crosstalk between cells in the tumor microenvironment, thus resulting in a non-effective cancer-related immune response. Altogether, these findings suggest the development of novel miRNA-based approaches directed to target immunomodulatory mechanisms. Reported data have also highlighted that miRNAs can exert their function in a cell type context-dependent manner. Thus, the design of more effective in vivo strategies of miRNA delivery needs to be improved, in particular focusing on enhancing tissue and cell specificity of miRNA vectors. Accordingly, exosome- and immune cell-based delivery represent two interesting potential strategies for miRNA-based cancer immunotherapy. In addition, the surface of carrier vesicles can be specifically modified with ligands or antibodies which are able to bind to the endogenous receptors of tumor or stromal cells. Therefore, the characterization of the tumor microenvironment in terms of miRNA/mRNA expression and their localization at cellular level is crucial. In addition, the mechanisms of miRNA-based modulation of immune responses need to be investigated in relation to different immunomodulatory therapies. In this context, the combination of miRNA-related immunotherapy with conventional cytotoxic drug agents or targeted therapy could represent a valuable opportunity for effective therapeutic interventions in human cancer.

## Abbreviations

CAF, cancer-associated fibroblast; CCL2, chemokine (C-C motif) ligand 2; CCL5, chemokine (C-C motif) ligand 5; CTL, CD8+ T cytotoxic cell; EGFR, epidermal growth factor receptor; ESCRT, endosomal sorting complex required for transport; EV, extracellular vesicle; EXP5, exportin 5; HCC, hepatocellular carcinoma; IKK, IkB kinase; IL6, interleukin 6; mRNA, messenger RNA; miRNA, microRNA; NFkB, nuclear factor kappa-B; NK, natural killer; NKG2D, NK group 2 member D receptor; NKT, natural killer T cells; pre-miRNA, precursor-miRNA; pri-miRNA, primary-miRNA; RISC, RNA-induced silencing complex; RUNX1, Runt-related transcription factor 1; STAT3, transducers activator of transcription 3; TAM, tumor-associated macrophage; TIL, tumor-infiltrating lymphocytes; TFH, T follicular helper cell; TH1, T helper 1 cells; TH2, T helper 2 cells; TH17, T helper 17 cells; TLR, Toll-like Receptor; TREG, regulatory T cells; UTR, untranslated region
